# What limits the utilization of health services among the rural population in the Dabie Mountains- Evidence from Hubei province, China?

**DOI:** 10.1186/1472-6963-14-379

**Published:** 2014-09-10

**Authors:** Pengqian Fang, Shilong Han, Lu Zhao, Zi Fang, Yang Zhang, Xiaoxu Zou

**Affiliations:** The College of Medicine and Health Management, Tongji Medical College, Huazhong University of Science and Technology, 13 Hangkong Road,Qiaokou District, Wuhan, Hubei Province 430030 P.R. China; College of Foreign Languages and Literature, Wuhan University, Wuhan, Hubei Province 430072 P.R. China; Tongji Hospital, Tongji Medical College, Huazhong University of Science and Technology, Wuhan, 430030 P.R. China

**Keywords:** Health service utilization, Economic status, Mountain areas, China

## Abstract

**Background:**

Individuals living in rural mountain areas tend to use health services less to manage discomfort or illness. This study aims to identify the variables that best explain the health service utilization of a sample of the rural population in the Dabie Mountains in China.

**Methods:**

To obtain information about health service utilization, a cross-sectional household survey was conducted using face-to-face interviews among the residents of a poor town in the Dabie Mountains. A total of 1,003 residents aged 15 or more, who had felt unwell in the last two weeks before the survey, were included in the analysis. The χ^2^ test and binary logistic regression were used to analyze the factors influencing health service utilization.

**Results:**

A total of 51.2% of those surveyed had not used health services when they felt unwell, higher than the data reported in the 4th National Health Services Survey of China. Enabling variables played an important role in predicting the utilization of health services. Factors associated with increased health service utilization included being younger, travelling longer to the nearest clinic, and higher household net income.

**Conclusion:**

To reduce disparities in health service utilization, (1) some effort should be made to change the perceptions and attitudes of older people; (2) reimbursement levels of the New Rural Cooperative Medical Care System should be improved to reduce economic barriers to health service utilization.

## Background

The utilization of health services has been found to be influenced by age [1], gender[1], physical disability [2], insurance status [3], economic situation [4–6], education [7], and social status [4]. Accessibility also plays a vital role in whether individuals will use health services [8]. Accessibility was defined as the relationship between the location of supply and that of clients, taking into account client transportation resources and travel time, distance and cost [9]. The key factors of inequitable accessibility are socioeconomic status, transportation, and geography [6, 10].

Despite improvements in health care accessibility accomplished by a series of medical and healthcare reforms, disparities remain, especially between urban and rural areas. One of the main goals of Chinese Healthcare Reform is to create equitable access to healthcare resources. Like many other countries, China is experiencing a rapid increase in relative income inequality, and also a significantly inequitable distribution of healthcare resources [11]. China is a vast country with a population of approximately 1.37 billion, of which 50.3% are spread across more than 700,000 rural and remote communities with limited health resources [12, 13].

Poorer rural communities have higher mortality than urban areas [14], increased financial burden caused by chronic diseases [15, 16], limited financial reimbursement for healthcare costs [16], a higher percentage of people at the poverty level [17], demographic aging [18], and de-population [19]. Health service providers in rural areas struggle to maintain primary health services because they lack financial support, infrastructure is not fit for purpose and they suffer from shortages of workforce, especially nurses [13, 20], which is associated with higher rates of patient mortality [21]. Misdistribution is arguably the most critical workforce challenge for universal coverage and for addressing inextricably-linked workforce problems such as shortages and skill imbalances [22, 23]. The ‘brain drain’ from rural to urban areas is a long-standing phenomenon in the health professions. In China, in 2011, urban areas had 7.9 health staff per 1,000 population, whereas rural areas with higher burdens of disease had only 3.19 health staff per 1,000 population [24]. Rural communities in mountain areas with high altitude face even more difficulties. Inhabitants living in mountain areas suffer a higher morbidity of some specific syndromes connected with hypoxia and hyperbaria, such as acute mountain sickness, and pulmonary and cerebral edema [25]. In addition, there are few public health campaigns in rural mountain areas. Without much opportunity and time to learn about health, residents of rural areas tend to lack awareness and health literacy. The difficulties of travelling to clinics make it harder for individuals to maintain their health, and they tend to visit health services only irregularly.

Studies of health service accessibility are common around the world. Despite a large number of studies focusing on health service accessibility in rural areas, little attention has been given to that in mountain areas, especially in China, partly because of a lack of data. The purpose of this study was therefore to fill this gap by exploring the health accessibility status and factors best explaining the unmet health needs of inhabitants of a rural and mountainous area of China.

## Methods

A cross-sectional rural household survey was conducted during July and August 2011, in Shengli, a poorer Chinese town located in Hubei province, in the Dabie Mountains, with altitudes from 500 to 1,700 meters. The Dabie Mountains are one of the largest mountain ranges in China, located at the junction of Anhui, Hubei, and Henan provinces. The study population was families living in the town. Shengli was chosen as the study site because it is one of the key towns for poverty alleviation and development in China. The economic situation in Hubei province, which is located in the middle of China, mirrors the rest of the country.

A multiphase stratified random sampling method was adopted to select the study sample, which covered seven administrative villages and 1,274 households. Families living in the same house were considered to be one household even if they were economically independent. For each village, two thirds of the households were selected randomly by house number. The numbers of households selected from the seven villages were therefore 198, 155, 183, 165, 179, 202 and 192. The study questionnaire was based on the National Health Services Survey designed by the Ministry of Health. Only questions relevant to this study were selected, including the household situation, demographic characteristics of family members, health status, and service utilization. Other questions such as accommodation condition and facilities, and specific information about children and pregnant women, were excluded. Trained graduate students from the School of Medicine and Health Management, Huazhong University of Science and Technology, conducted the in-home interviews in Chinese.

A face-to-face questionnaire survey was conducted from house to house with individuals who were at home. For those household members absent at the time of the interview, a total of 6.8%, the head of the household provided the information. All such information was assumed to be reliable. Members of 1,248 households were interviewed and the response rate was 98.0%. After each interview, which lasted approximately 15 minutes, the participant received a small token of thanks (a towel or a comb). A total of 1,193 residents reported having experienced physical discomfort or illness in the previous two weeks, of which 190 were excluded because they were under 15 years old. The total number of residents included in the analysis was 1,003.

The model of health services utilization used in this study was that developed by Andersen [8, 26]. It was originally developed to understand the social, individual, and systemic factors that influenced health service use. The model suggested that the inclination of individuals to use health services could be explained by several factors including predisposing factors (factors which existed prior to illness), enabling factors (the availability and accessibility of health resources), and need variables (both perceived and clinician-evaluated).

Data were entered into EpiData Info version 3.1 databases. PASW Statistics 18.0 (formerly SPSS) was used for statistical analysis. The characteristics of study variables were summarized by using descriptive statistics. The analysis was divided into two parts. In the first part, a χ^2^ test with a minimum confidence level of *p* < 0.05 was used to establish the existence of associations for each variable and health service utilization. Variables that were statistically significant were selected for further analysis, in which a binary logistic regression analysis with a minimum confidence level of *p* < 0.05 was used to identify the most important factors affecting health service utilization by comparing health service users and non-users. Dependent and independent variables are shown in Table 1.

**Table 1:** **Description of dependent and independent variables**

***Dependent variables***	***Health service utilization***
*Independent variables*	
*Predisposing variables*	Gender, age, marital status, level of education, and job category
*Enabling variables*	Household size, medical insurance, transportation, travel time, and income
*Need variables*	Number of chronic illnesses diagnosed

Respondents were asked if they had used any health services as a result of any problem, discomfort, or illness within the last two weeks. Users were coded as “1” and non-users as “0”. For need variables, self-reported health status and number of chronic illnesses diagnosed were included during the investigation. However, self-reported health status was not included in the analysis because information was not available in 25% of the cases.

### Ethics statement

Ethical approval has been obtained from the Ethics Committee of Tongji Medical College, Huazhong University of Science and Technology (IRB No: FWA00007304), and informed consent was obtained from the 3,809 participants.

## Results

### Sample characteristics

The final sample was 42.5% male, and nearly half (46.1%) reported that they were between the ages of 45 and 60. Around one third, 33.5% of the sample, had received only primary education. The mean household size was 3.57 people. 1.4% of the sample had medical insurance other than the New Rural Cooperative medical care, which was universal (95.8%) in rural areas. Although 49.3% reported that the distance from their home to the nearest village clinic was less than one kilometer, 7.8% had to travel more than three kilometers. A large proportion, 85.3%, of the sample reported that the easiest way to travel to the village clinic was walking, and 47.0% said they could reach the clinic within 30 minutes. Around a third, 30.9%, reported an annual income of less than RMB5000. Table 2 shows the detailed demographic information.

**Table 2:** **Characteristics of study variables and univariate analysis examining factors associated with health service utilization**

Variables	Total N (%)	Health service utilization		P
		Yes	No	
All the residents	1003(100)	489(48.8)	514(51.2)	
Gender				0.026
Male	426(42.5)	192(45.1)	234(54.9)	
Female	577(57.5)	297(51.5)	280(48.5)	
Age				<0.001
15–30 years	79(7.9)	53(67.1)	26(32.9)	
30–45 years	187(18.6)	116(62.0)	71(38.0)	
45–60 years	462(46.1)	206(44.6)	256(55.4)	
> = 60 years	275(27.4)	114(41.5)	161(58.5)	
Marital status				0.379
Unmarried	173(17.2)	82(47.4)	91(52.6)	
Married	830(82.8)	407(49.0)	423(51.0)	
Level of education				0.181
< primary school graduate	336(33.5)	154(45.8)	182(54.2)	
< high school graduate	556(55.4)	273(49.1)	283(50.9)	
Higher studies	111(11.1)	62(55.9)	49(44.1)	
Job category				0.03
Agricultural workers	797(79.5)	376(47.2)	421(52.8)	
Non-agricultural workers	206(20.5)	113(54.9)	93(45.1)	
Household size				0.003
1 person	91(9.1)	45(49.5)	46(50.5)	
2 persons	347(34.6)	146(42.1)	201(57.9)	
3 persons	233(23.2)	108(46.4)	125(53.6)	
4 persons	187(18.6)	108(57.8)	79(42.2)	
> = 5 persons	145(14.5)	82(56.6)	63(43.4)	
Medical insurance				0.095
Uninsured	28(2.8)	8(28.6)	20(71.4)	
New rural cooperative medical care system(NCMS)	961(95.8)	474(49.3)	487(50.7)	
Other kind of insurance	14(1.4)	7(50.0)	7(50.0)	
Transportation media				0.247
Walking	856(85.3)	413(48.2)	443(51.8)	
Communication media	147(14.7)	76(51.7)	71(48.3)	
Travel time				0.022
<30 minutes	471(47.0)	216(45.9)	255(54.1)	
30–60 minutes	423(42.2)	207(48.9)	216(51.1)	
>60 minutes	109(10.8)	66(60.6)	43(39.4)	
Household net income per year				0.009
<RMB5000	310(30.9)	140(45.2)	170(54.8)	
RMB5000–10000	297(29.6)	142(47.8)	155(52.2)	
RMB10000–20000	127(12.7)	55(43.3)	72(56.7)	
RMB20000–40000	154(15.3)	79(51.3)	75(48.7)	
> = RMB40000	115(11.5)	73(63.5)	42(36.5)	
Number of chronic illnesses				0.143
0–1	560(55.8)	277(49.5)	283(50.5)	
1–2	329(32.8)	151(45.9)	178(54.1)	
2–3	92(9.2)	53(57.6)	39(42.4)	
>2–4	22(2.2)	8(36.4)	14(63.6)	

(n = 1,003).

### Univariate analysis

A χ^2^ test was conducted to test the differences among selected variables. Table 2 shows the predisposing variables. Gender (*p* = 0.026) and age (*p* < 0.001) were significantly associated with the use of health services, with women and older people being more likely to use health services when they felt unwell. Although slightly more health service users were married and had received higher education, neither factor was significantly associated with use of health services.

Household size (*p* = 0.003) was associated with health service use, with a larger size contributing to higher service utilization. Slightly higher numbers of those who were uninsured chose not to use health services, but medical insurance status had no statistical association with health service utilization. The time needed to travel to the nearest village clinic (*p* = 0.022) was significantly associated with the use of health services. Residents who had to travel longer were more likely to seek medical help, although mode of transport was not significant. Household net income (*p* = 0.009) was significantly associated with health service utilization; those with higher incomes were more likely to use health services. The number of chronic illnesses diagnosed had no relationship with health service use.

### Multivariate analysis

A binary logistic regression model was constructed to identify the variables that best explained service use by people who felt unwell in the two weeks before the survey. To select meaningful variables for regression, only those found to be significant using the χ^2^ test were included. Age, household size, travel time and household income were entered as dummy variables.

Table 3 shows the regression model, which was significant (χ^2^ = 67.088, *df* = 15, *p* < 0.001). Results from the multivariate analyses indicated that age (*p* < 0.001), travel time (*p* = 0.006), and household income (*p* = 0.034) significantly distinguished participants who used health services from those who did not. Older people were less likely to use health services when they felt unwell. Those between the ages of 15 and 30 (OR = 2.757, 95% CI = 1.588–4.784, *p* < 0.001) and between the ages of 30 and 45 (OR = 2.122, 95% CI = 1.401–3.216, *p* < 0.001) were more likely that those over 60 to use health services. Residents who had to travel further were more likely to use health services when they felt unwell. Those with a travel time of less than 30 minutes (OR = 0.488, 95% CI = 0.313–0.761, *p* = 0.0021) and between 30 to 60 minutes (OR = 0.601, 95% CI = 0.385–0.937, *p* = 0.025) were less likely to use health services than those who had to travel for more than one hour. Residents with higher household income were more likely to use health services. Those with a household income of less than RMB5,000 (OR = 0.532, 95% CI = 0.330–0.860, *p* = 0.010), RMB5,000 to 10,000 (OR = 0.544, 95% CI = 0.342–0.864, *p* = 0.010), and RMB10,000 to 20,000 (OR = 0.435, 95% CI = 0.254–0.744, *p* = 0.002) were less likely to use health services than those with an income of more than RMB40,000. There were no interaction effects between income and distance and age and distance (Table 4). Table 5 shows why the participants did not use health services when they felt unwell. The most significant reasons given were financial difficulty (37.9%), lack of need (27.2%), and self-medication (19.8%).

**Table 3:** **Logistic regression model predicting health service utilization**

Variables	Reference category	B	Odds Ratio (95% Confidence intervals)	P value
Gender		0.183	1.201(0.918–1.570)	0.181
Job category		-0.059	0.943(0.848–1.047)	0.272
Age	> = 60 years			<0.001
15–30 years		1.014	2.757(1.588–4.784)	<0.001
30–45 years		0.753	2.122(1.401–3.216)	<0.001
45–60 years		0.103	1.109(0.806–1.526)	0.526
Household size	> = 5 persons			0.076
1 person		-0.005	0.995(0.559–1.771)	0.987
2 persons		-0.368	0.692(0.453–1.058)	0.089
3 persons		-0.354	0.702(0.452–1.090)	0.115
4 persons		0.072	1.075(0.677–1.707)	0.760
Travel time	>60 minutes			0.006
<30 minutes		-0.718	0.488(0.313–0.761)	0.002
30–60 minutes		-0.510	0.601(0.385–0.937)	0.025
Household income	> = RMB40000			0.034
<RMB5000		-0.631	0.532(0.330–0.860)	0.010
RMB5000–10000		-0.609	0.544(0.342–0.864)	0.010
RMB10000–20000		-0.833	0.435(0.254–0.744)	0.002
RMB20000–40000		-0.482	0.618(0.370–1.032)	0.066

**Table 4:** **Interaction effects between income and distance and age and distance**

	B	P	Exp(B)		95% C.I.for EXP(B)
				Lower	Upper
GENDER	0.181	0.178	1.198	0.921	1.559
AGE	-0.560	0.005	0.571	0.386	0.844
JOB	-0.042	0.416	0.958	0.865	1.062
SIZE	0.086	0.135	1.090	0.974	1.220
TIME	-0.137	0.788	0.872	0.322	2.363
INCOME	0.158	0.211	1.171	0.915	1.499
INCOME by TIME	-0.034	0.621	0.966	0.843	1.107
AGE by TIME	0.136	0.230	1.146	0.917	1.431
Constant	1.041	0.302	2.831

**Table 5:** **Self-reported reasons for not using health services**

Reasons	Non-users	
	n	%
Not necessary	140	27.2
Financial difficulty	195	37.9
Have no time	24	4.7
Traffic inconvenience	13	2.5
No effective measures	40	7.8
Self-medication	102	19.8

## Discussion

This study aimed to explore the factors influencing health service utilization among residents of the Dabie Mountains in China. A previous study [27] showed that living at higher altitude decreased the likelihood of people seeking healthcare. Our results demonstrate that 51.2% of people did not use health services despite feeling unwell within the two weeks before the survey. These findings are higher than the 35.6% in rural areas reported in the 4th National Health Services Survey, conducted every five years by the Ministry of Health.

The main reason why non-users did not seek help was financial difficulty, which accounted for 37.9% in our survey, but only 14.9% in the 4th National Health Services Survey. Results from the regression analysis indicate that residents with higher household net income per year were more likely to use health services, which indicates that the economic factors affecting health services use may be more important among those living in mountain areas than in the rest of the country. For most rural residents, more farmland means a higher income, but those living in mountain areas tend to have less farmland because of the terrain. The level of income in mountain areas is therefore lower than on the plains. This situation has decreased the financial affordability of healthcare. Being poor because of illness is a very common problem in rural China.

Enabling variables play a larger role in predicting the utilization of health services, with two statistically significant variables. Of the predisposing variables, age was the only statistically significant factor that discriminated between users and non-users. Older residents were less likely to use health services when they felt unwell. The two most commonly reported reasons were lack of need and preference for self-medication, which indicated that possible cognitive appraisal might affect the behavior of older people. Studies have shown that cognitive appraisal significantly predicted mental health service utilization [28]. Women and non-agricultural workers were more likely to use health services in the univariate analysis, but this was not statistically significant in the multivariate analysis. Gender and job category might influence use of health services, but other factors such as age and travel time overshadowed these influences in this study. Marital status and level of education were not distinguishing factors between health services users and non-users in this study.

Of the enabling variables, travel time and household income were statistically significant in distinguishing health service users from non-users. Residents who had to travel further were more likely to use health services, which was different from previous studies in China or elsewhere. There were no statistically significant interaction effects between age and travel time (P = 0.230), or income and travel time (P = 0.621). Deen reported a similar conclusion that more environmental barriers predicted higher levels of health service use [28]. It may be that residents who had to travel further were living in higher altitude areas. Higher altitude is characterized by sustained hypoxia [25], which may result in serious medical problems, so that residents of those areas are more likely to seek help when they feel unwell. As the health services provided by the health center in the town are much better than those provided in the village clinic, people were more likely to go to the town’s health center for help. When health status gets worse, distance tends to matter less [3]. Thus, when residents decided to see a doctor, the travel time to the nearest clinic was not important, and these residents were willing to travel longer to seek help from a doctor or a therapist. Residents with higher household net income per year were also more likely to use health services.

Medical insurance status was a significant factor in the univariate analysis, but not in the multivariate analysis. In China, a rural health insurance system called the New Cooperative Medical Scheme (NCMS) was started in 2003. This is the main kind of insurance in rural areas and aims for universal health coverage for rural residents. Most (95.8%) of those surveyed were covered by the NCMS. Previous studies have shown that insurance does influence health service utilization [29]. However, this study found the insurance premiums paid by individuals were lower because of the economic barriers. Only a small part of the individual contribution was allocated to cover outpatient services. Lower insurance premiums paid by individuals and the lower outpatient reimbursement level of the NCMS result in high out-of-pocket costs. Outpatient service utilization did not significantly change under NCMS [30]. One previous study found that the two most prominent manifestations of irrational drug prescribing were overuse of injections and antibiotics in village health clinics [31]. This may also increase the economic burden to rural residents.

The number of chronic illnesses was not statistically significant in predicting health service use. However, this finding does not mean that need variables are unimportant, since this study only included one need variable. Other studies have shown that need variables play a far more important role than predisposing or enabling variables in predicting health service use [32].

### Limitation

This study has several limitations. First, the data were only from the Dabie Mountains, so the results may not be representative of other areas, mountainous or otherwise. Further research is needed in other mountain areas, and also other rural areas. Second, information about socioeconomic and health status was not enough. Ethnicity and evaluated health status, which were not included in the survey, might underestimate the health services use in mountain areas in China.

## Conclusion

In mountain areas, the percentage of residents who did not use health services despite feeling unwell appeared to be higher than elsewhere. Age and income were important barriers for non-users. Residents with longer travel time to the nearest clinic were more likely to use health services, which was different from previous studies. There was no significant difference between those who were insured and those who were not. Targeting the perceptions and attitudes of older people will be important to reduce the disparities in health service utilization. Improving reimbursement levels of the New Rural Cooperative Medical Care System could also reduce economic barriers.
